# Medication Exposure and Risk of Dementia and Alzheimer’s Disease

**DOI:** 10.3390/ijms252312850

**Published:** 2024-11-29

**Authors:** Niti Sharma, Seong Soo A. An, Sang Yun Kim

**Affiliations:** 1 Bionano Research Institute, Gachon University, 1342 Seongnam-daero, Sujeong-gu, Seongnam-si 13120, Republic of Korea; nitisharma@gachon.ac.kr; 2 Department of Neurology, Seoul National University Bundang Hospital & Seoul National University College of Medicine, 82, Gumi-ro 173 Beon-gil, Bundang-gu, Seongnam-si 13620, Republic of Korea

**Keywords:** alzheimer’s disease, dementia risk, anticholinergics, analgesics, benzodiazepines, proton pump inhibitors, statins

## Abstract

Alzheimer’s disease (AD), a complex neurodegenerative disease (ND), is the most predominant cause of dementia among the elderly. Generally, elderly people have multiple chronic health conditions, like hypertension, arthritis, diabetes, insomnia, bowel problems, and depression. Although prescribed medications have beneficial therapeutic compositions, some may have side effects that could hinder cognitive function or worsen cognitive decline. Hence, we should evaluate those medications to guarantee their safety. In the present mechanistic review, we discussed frequently used categories of medication (analgesics, anticholinergics, benzodiazepines, proton pump inhibitors, and statins), concerning their possible involvement in increasing AD and dementia risks. This review summarized the results of various observational studies, meta-analyses, randomized case–control studies, and systematic reviews. As the results were contradictory, it was difficult to ascertain the clear associations between medication usage and increased risks of dementia or AD. The blood-based biomarkers (BBMs) offer a low-cost and accessible alternative for early diagnosis of AD. Systematic reviews combined with meta-analysis would be crucial tools for accurately assessing and summarizing the efficacy of health interventions, yet randomized clinical trials have always been the best way to help with clinical care decisions. Thus, an open discussion is necessary to help individuals determine whether the advantages of utilizing medications outweigh the possible drawbacks.

## 1. Introduction

Alzheimer’s disease (AD) is a complex neurodegenerative disease (ND), associated with progressive decline in memory and cognition. AD is defined by distinct alterations in the brain, such as the accumulation of amyloid-beta (Aβ) protein and neurofibrillary tangles (NFTs) of phosphorylated tau (p-tau) proteins, leading to progressive neuronal loss. Nearly 6.9 million elderly in the United States (US) have AD with 0.2 million under 65 years old believed to have early-onset AD (EOAD). This number could increase to 13.8 million by 2060 [1]. Statistically, the healthcare expenditure and long-term care for individuals with AD have increased, reaching a projected total of USD 360 billion in 2024, reflecting a USD 15 billion rise from the previous year [2]. AD is the most common cause of dementia and may contribute to over 60% of the cases [3], followed by vascular dementia (VD), Lewy body dementia (LBD), frontotemporal dementia (FTD), and mixed-type dementia. Dementia is a syndrome marked by a gradual decline in cognitive and functional abilities significant enough to impact daily activities. With the rising life expectancies, the global population of individuals with dementia is predicted to increase. Globally, approximately 57 million individuals are impacted by dementia and this number is expected to increase to 153 million by 2050 [2].

As life expectancy increases due to better healthcare and lifestyle, the global population of individuals aged 65 years or older would increase with numbers set to surpass 1.6 billion by 2050, more than double the 761 million in 2021. The population of individuals 80 years and older will increase at an accelerated rate and is expected to triple by 2050 [4]. Generally, elderly people have multiple chronic health conditions, like hypertension, arthritis, diabetes, obstructive chronic pulmonary disease, insomnia, indigestion, bowel problems, and depression. As a result, they often consume a higher number of both prescription and non-prescription drugs and thus, they are prone to drug–drug interactions that may enhance adverse outcomes, like falls, injury, and delirium. Moreover, the organs responsible for detoxification also become less effective at this age, which may cause drug toxicity. The risk of potentially inappropriate medications (PIMs) administration would increase in polypharmacy. If these medications would affect cognition, their prolonged use could lead to dementia [5]. Recent research indicated that certain drugs, like anticonvulsants and anticholinergic medications, might be linked to a higher likelihood of developing dementia. This suggested that by avoiding these medications, the risk of developing dementia could potentially be reduced. Yet, it will be frequently challenging to determine the factors contributing to cognitive alterations in the elderly using polypharmacy that can independently impact cognitive abilities. It would be crucial to closely monitor the risks of AD and dementia by addressing modifiable risk factors and assessing through various blood-based biomarkers to prevent dementia on a worldwide scale effectively.

The presence of extensive healthcare data from large populations, along with advancements in computational power, allowed the systematic investigation into the link between medications and diseases. As a result, several meta-analyses, observational studies, cohort studies, and clinical studies were carried out on several suspicious drugs for increasing AD and dementia risks. Still, a comprehensive review of the studies’ outcomes would be required. Therefore, in this review, we searched and summarized the existing literature and discussed various classes of drugs that have been associated with the risk of dementia and AD. We anticipate that this review will be helpful to clinicians, researchers, and policymakers and assist in best prescribing guidelines for medicines for preventing and treating patients to reduce AD and dementia.

## 2. Categories of Drugs Linked to Increased Risks of Dementia and AD

According to the literature, the use of certain medications, such as analgesics, anticholinergics, and benzodiazepines, could be associated with varying risks for developing AD and other forms of dementia [6,7,8]. On the other hand, PPIs and statins have unclear or varied connections to dementia, with some indications of potential protective effects or minimal risks [9,10]. The elderly (60+ years) tended to have higher usage rates in comparison to young adults due to age-related health conditions and the prevalence of chronic diseases. Table 1 presents the general breakdown of their use, though exact percentages may vary based on country, healthcare system, and population demographics.

For a drug to affect brain function, it must reach the brain by crossing the blood–brain barrier (BBB), which depends on several criteria including lipophilicity. Analgesics like ibuprofen and naproxen have low permeability [23], whereas anticholinergics and benzodiazepines show powerful penetration through the BBB for their therapeutic effect [24,25]. Proton pump inhibitors and statins have different levels of permeability, indicating the possibility of both positive and negative effects in brain-related disorders [26,27]. This comprehension is crucial for improving drug development and therapeutic approaches in central nervous system disorders. Brief information on the targets, protein expression, and location of the targets has been provided (Table 2).

### 2.1. Analgesics

**Figure 1:**
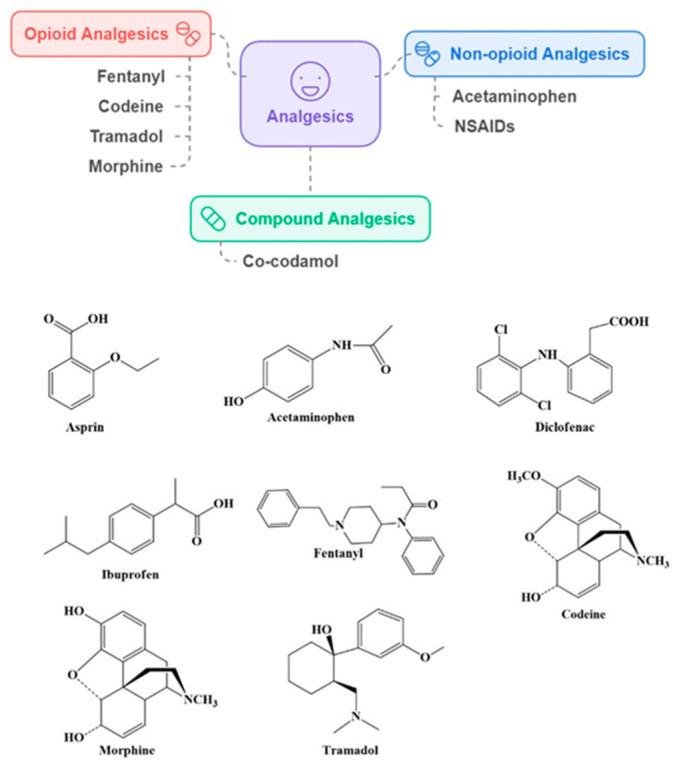
Broad classification and structures of some analgesics.

Analgesics are drugs employed for the control and therapy of pain. They function by either diminishing inflammations or altering the brain’s processing and perceptions of pain. Significant global use of pain relievers was driven by the common need for managing various types of pains, such as headaches, muscle pain, injuries, chronic pain, and post-operative recovery. The global analgesics market generated USD 91.62 billion in 2024 and is expected to fetch USD 143.65 billion by 2034 [28]. Over-the-counter (OTC) pain relievers, like paracetamol (acetaminophen), ibuprofen, and aspirin, were among the most widely consumed medications with a global market size of USD 28.6 billion in 2022, which is expected to grow USD 40.9 billion by 2032 [29]. Analgesics have been broadly categorized as non-opioid analgesics (acetaminophen and nonsteroidal anti-inflammatory drugs: NSAIDs, e.g., aspirin, diclofenac, ibuprofen), compound analgesics (combination of non-opioid and weak opioids, e.g., co-codamol a combination of paracetamol and codeine), and opioid analgesics (e.g., fentanyl, codeine, tramadol, morphine) (Figure 1). Regular to high doses of non-aspirin traditional NSAIDs could lead to serious adverse effects on the gastrointestinal and cardiovascular systems.

Opioids function by stimulating opioid receptors in both the central and peripheral nervous systems, which decreases the activity of neurons, resulting in a decrease in the transmission of pain signals. Opioid pain medications, such as morphine, oxycodone, and tramadol, were frequently prescribed in healthcare environments to manage intense or prolonged pain, particularly for ailments, like cancer or after surgical procedures. A study indicated that 84% of elderly (62–86 years) were on prescription analgesic medications, among which NSAIDs were the most common (77%), followed by paracetamol (41%), and opioids (32%). This status showed a significant usage of painkillers and their quantities in elderly people, underscoring the necessity of overseeing and controlling pain in this population with opioids [11].

#### Association of Analgesics with Dementia and AD Risk

Opioids may cause cognitive decline by affecting various cognitive functions in the mu (μ)- and kappa (κ)-opioid receptor systems. They could impact primarily hippocampal neurons by boosting glutamate release in presynaptic neurons and enhancing the activities of postsynaptic receptors. The presence of opioids in a synapse prevented the blockage of the N-methyl-D-aspartate receptor (NMDAR) by Mg^2+^ and increased the NMDAR conductance, eventually inducing long-term potentiation (LTP) [30]. Opioids might change the levels of neurotransmitters, which could impact cognitive function [31,32], and have also been reported to reduce neurogenesis [33].

Individuals on opioids or NSAIDs had slightly increased risks of dementia in comparison to those with less frequent usage or none. In a prospective study (*n* = 194,758) from the UK Biobank, individuals were grouped into different painkiller categories. In comparison to those without NSAIDs, the aspirin, paracetamol, and 2–3 NSAIDs groups had a greater risk of all-cause dementia (ACD) (Hazard ratio [HR]: 1.12, 1.15 and 1.2, respectively, 95% confidence interval [CI]: 1.01–1.24, 1.05–1.27 and 1.08–1.33, *p* < 0.05 for aspirin and 2–3 NSAIDs, *p* < 0.01 for paracetamol). The ibuprofen group did not exhibit a noteworthy correlation with the likelihood of ACD, AD, or VD [34]. Frequent use of paracetamol was also linked to an increased chance of developing dementia in older individuals, irrespective of genetic predisposition to the disease [35]. Extended use of aspirin has been associated with gastrointestinal bleeding, which may result in anemia and reduced blood supply to the brain, leading to cognitive decline [36,37]. Aspirin blocks cyclooxygenase-2 (COX-2), an enzyme involved in the synthesis of prostaglandin E2 (PGE2), a neurotransmitter managing synaptic plasticity and memory [38]. Paracetamol is converted to a toxic metabolite named N-acetyl-p-benzoquinoneimine (NAPQI) in the liver, leading to a decrease in glutathione levels and causing oxidative stress in the body [39].

**Figure 2:**
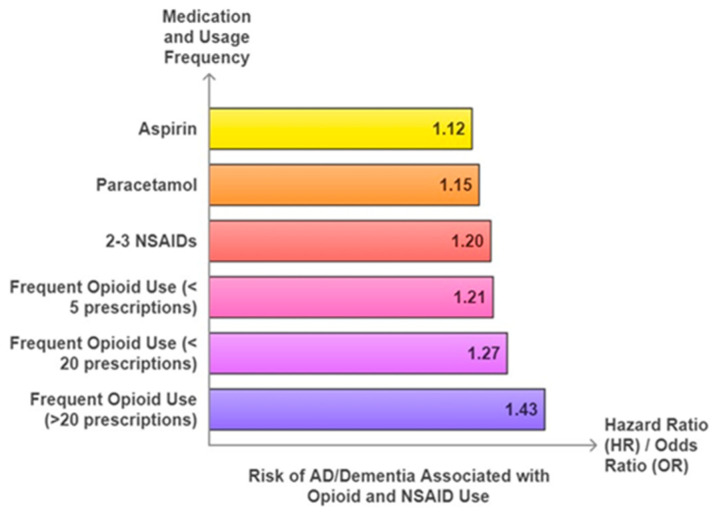
Increased risk of AD/dementia associated with the use of analgesics. Frequent use of opioids (>20 prescriptions) was associated with the highest risk of AD/dementia in comparison to NSAIDs. The graph was plotted using data reported by Gao et al., 2024 [43].

Recently, a population cohort-based study was conducted on 1,261,682 Korean patients suffering from chronic non-cancer pain (CNCP), 21,800 of whom were opioid users [40], which demonstrated the increased risks of dementia (15%), AD (15%), and unspecified dementia (16%) in adults with CNCP on opioids in comparison to the control group. However, no significant association was observed for VD. A nationwide study in Israel discovered that the elderly population (75 to 80 years) on opioid drugs had a 1.39-fold increased likelihood of developing dementia (aHR: 1.39, 95% CI: 1.01–1.92, *p* < 0.05) in comparison to non-users [41]. In another population-based study of diagnosed participants aged 65 and older with dementia, 42% were on opioid therapy [42]. The nested case–control study on UK Biobank participants with chronic pain and regular analgesic usages indicated increased opioid use in correlation with an escalated risk of dementia (1 to 5 prescriptions: Odds ratio [OR]: 1.21, 95% CI: 1.07–1.37, Wald χ^2^: 3.02, degrees of freedom (df):1, *p*: 0.003; 6 to 20: OR: 1.27, 95% CI: 1.08–1.50, Wald χ^2^: 2.93, df:1, *p*: 0.003; more than 20: OR: 1.43, 95% CI: 1.23–1.67, Wald χ^2^: 4.57, df: 1, *p* < 0.001) [43] (Figure 2).

Among participants in the adult with changes in thought (ACT) study, elevated NSAID consumptions were linked to higher chances of being clinically diagnosed with AD dementia (HR: 1.55, 95% CI: 1.07–2.24) [44] and elevated neuritic plaque (NP) scores (Relative risk [RR]: 2.37; 95%, CI 1.24–4.67) [45]. Although opioid use had a slight association with dementia (HR:1.29, 95%; CI: 1.02–1.62), it was not associated with greater neuropathologic changes (higher NP or NFT scores) [46]. Studies concluded that participants with persistent exposures to NSAIDs had a higher likelihood of developing AD (HR:1.57; 95%; CI:1.10–2.23) and dementia (HR:1.66; 95%; CI:1.24–2.24) [44]. In addition, increased NSAID usages were correlated to higher levels of Aβ42 in the middle frontal gyrus (MFG) and superior and middle temporal gyri (SMTG) regions, but not to increased p-tau levels in any of the above areas. On the other hand, increased opioid usage correlated with higher p-tau levels in the MFG specifically, but did not correlate with Aβ42 levels in any brain region in comparison to those with minimal or no opioid usage. Additionally, frequent opioid usage was linked to the decreased grey matter and hippocampal volumes, as well as the increased white matter hyperintensity volumes. The variation in results could be explained by different mechanisms of action of the two drugs [47].

On the other hand, considerable data indicated that NSAID drugs may delay or even stop the development of AD in other studies [48,49,50,51,52]. A meta-analysis of 17 epidemiological studies found a negative association between the use of NSAIDs and AD risk in individuals from eight countries [49]. AD lesions are defined by persistent neuroinflammation that could lead to the destruction of neurons. The anti-inflammatory drugs delay the onset of inflammation, helping delay AD progression. The probable mechanisms involve the activation of the peroxisome proliferator-activated receptor gamma (PPARγ) and inhibition of COX and β-secretase gene promoter activities by NSAIDs [48,53,54], suppressing the formation of amyloid plaques and inflammatory substances. In a systematic review and meta-analysis of observational studies (*n* = 13,211), the risk for short-term users (<1 month) was 0.95 (0.70–1.29), while the risk for the intermediate-term users (<24 months) was 0.83 (0.65–1.06) and 0.27 (0.13–0.58) for long-term users (>24 months). The combined relative risk among the eight studies with aspirin users was 0.87 (0.70–1.07) [52]. The relative risk (RR) for AD decreased with the increased duration of NSAID usage [RR: 0.40 (>2 years); RR: 0.65 (<2 years)] [50]. In a prospective cohort study [55], no association was found between the use of prescription opioids (HR: 1.29, 95% CI: 1.02–1.62) or NSAIDs (HR: 1.31, 95% CI: 1.07–1.62) and increased risks of cognitive decline or dementia. Individuals who used high doses of opioids did not show a faster decrease in mental performance upon aging [55]. Likewise, in a Finnish nationwide nested case–control study on a Medication use and Alzheimer’s disease (MEDALZ) cohort (*n* = 70,718), no link was found between opioid use and a higher risk of AD (aOR: 1.00, 95% CI: 0.98–1.03). Extended use of opioids (>365 days: aOR: 1.02, 95% CI: 0.96–1.08) or high cumulative doses (>90 total standardized doses (TSDs): aOR: 1.02, 95% CI: 0.98–1.07) did not increase the risks of AD [56]. Furthermore, no dementia-like pathology was observed in the community-based autopsy cohort (*n* = 420) for opioids or NSAID users [46]. A recent systematic review and meta-analysis from 16 cohort studies (*n* = 236,022) revealed a significant link between using NSAIDs and a decreased risk of AD, in comparison to those without using NSAIDs (RR: 0.81, 95% CI: 0.70 –0.94) [57]. The results varied based on the geographical location; a significant association was observed with the European population (RR: 0.72, 95% CI: 0.56–0.92); a modest association with the North American population (RR: 0.87, 95% CI: 0.72–1.06), while no association in the Asian group (RR: 0.87, 95% CI: 0.36–2.10) was observed in the context of reduced risk of AD [57]. Moreover, prolonged use of NSAIDs could lower the risk of developing AD, but do not provide the same protection against VD [58]. A summary of related studies is presented in Table 3.

**Figure 3:**
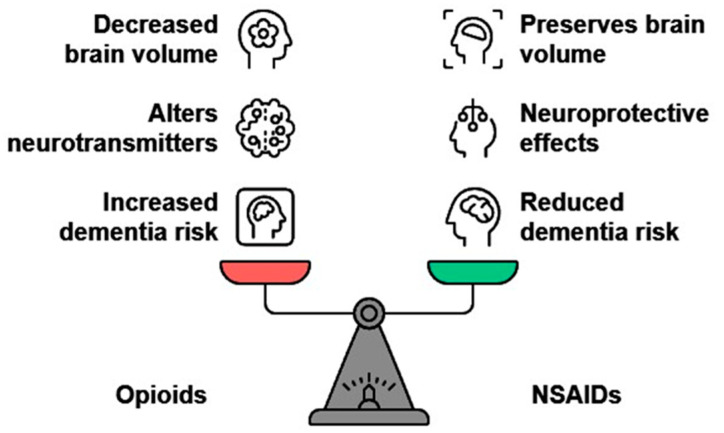
Evaluating cognitive impacts of opioids vs. NSAIDs.

Hence, the above pieces of evidence highlighted the complex relationship between opioid and NSAID usage and cognitive decline, particularly in the context of dementia risks. While opioids may contribute to cognitive impairment through neurobiological mechanisms, NSAIDs appeared to have a protective effect against dementia from extended usage. As far as analgesics were concerned, the reported literature was contradictory and did not provide conclusive evidence for any association between analgesic usage and increased risks of AD/dementia. Further research is required to clarify these associations and comprehend the underlying mechanisms (Figure 3).

### 2.2. Anticholinergic Drugs

**Figure 4:**
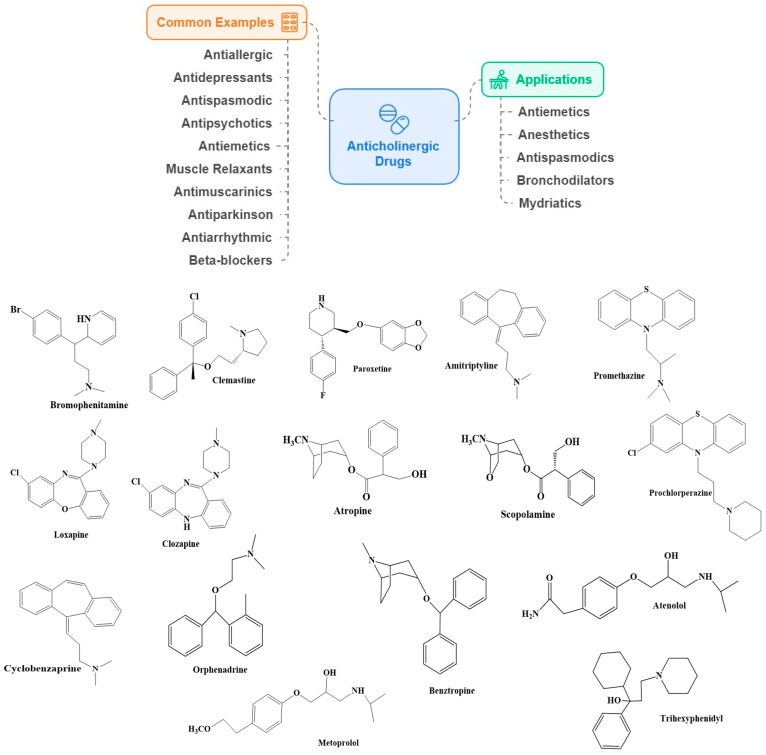
Some common examples and applications of anticholinergic drugs.

Anticholinergics can cross the BBB and block neurotransmitter acetylcholine (ACh) actions at synapses in the central and peripheral nervous systems (CNS and PNS). Based on the target, drugs have been divided into antimuscarinic and antinicotinic agents. Antimuscarinic agents inhibit ACh binding to muscarinic receptors (mAChR) without affecting nicotinic receptors at the neuromuscular junction, while antinicotinic agents target the nicotinic acetylcholine receptors (nAChR). The majority are non-depolarizing skeletal muscle relaxants used in surgery. Medications with anticholinergic activity impacted mAChR mainly. Anticholinergics have a spectrum of therapeutic applications, like antiemetics, anesthetics, antispasmodics, bronchodilators, and mydriatics [59]. Several commonly used anticholinergics are antiallergic (brompheniramine, clemastine), antidepressants (amitriptyline, paroxetine), antispasmodic (atropine, scopolamine); antipsychotics (clozapine, loxapine), antiemetics (prochlorperazine, promethazine), muscle relaxant (cyclobenzaprine, orphenadrine), antimuscarinics (flavoxate, oxybutynin); anti-Parkinson’s (benztropine, trihexyphenidyl), and antiarrhythmic (disopyramide); blood-pressure lowering medicine (beta (β)-blockers like atenolol, metoprolol) (Figure 4).

Elderly people with multiple health conditions have been commonly given medications with anticholinergic effects, which are often used to treat various conditions like insomnia, chronic obstructive pulmonary disease (COPD), depression, Parkinson’s disease (PD), allergies, and overactive bladder. Anticholinergic burden (ACB) is the term used to describe the increased risk of medication-related adverse effects caused by the accumulation of higher levels of exposure to one or more anticholinergic medications. The ACB scale has been frequently used to classify anticholinergics. Drugs with ACB scores of 3 (e.g., atropine and flavoxate) had strong anticholinergic properties in comparison to drugs with a score of 2 (moderate; e.g., cyclobenzaprine and loxapine), and 1 (mild; e.g., atenolol and codeine). Typically, 64.3% of older individuals used anticholinergics, while 9.9% used strong anticholinergics [60]. The common side effects of these drugs include confusion and memory loss, especially in the elderly [61].

#### Association of Anticholinergics with Dementia and AD Risk

**Figure 5:**
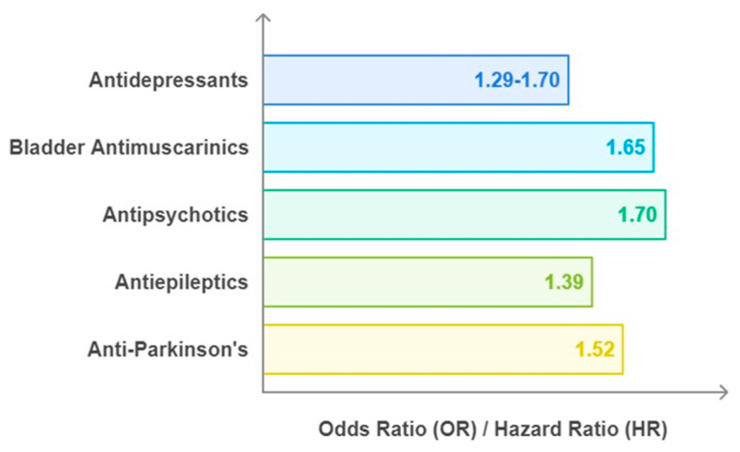
Association between anticholinergic use and AD/dementia risk. In this category, antipsychotics pose a higher risk of AD/dementia in comparison to others. The graph was plotted using data reported by Coupland et al., 2024 [70].

Anticholinergic drugs may cause short-term cognitive side effects [61,62] and it was generally believed that cognitive impairment could be reversed by stopping the medication [63]. Nonetheless, multiple reports indicated that anticholinergic drugs could potentially lead to a higher likelihood of long-term cognitive issues [64,65,66]. To ascertain whether prolonged usages of these medications were associated with a higher risk of developing dementia, several studies were conducted as follows [64]. In a German cohort study, 2605 elderly (age > 75 years) without dementia were followed for 4.5 years after starting anticholinergic drugs, revealing a doubled risk of developing dementia (HR: 2.08; 95%). There was a substantially higher risk of developing dementia (HR:1.54) and AD (HR: 1.63) in patients with the greatest anticholinergic burden in comparison to those not taking such medication [7]. Several observational studies were conducted to prove a relationship between anticholinergics and dementia risks [67,68,69]. In a nested case–control study of 58,769 patients, Coupland et al. [70] discovered a higher risk of dementia linked to the cumulative use of anticholinergic medication in a large (284,343 case patients and matched controls) nested case–control study. The antidepressants (aOR: 1.29; 95% CI: 1.24–1.34), bladder antimuscarinics (aOR: 1.65; 95% CI: 1.56–1.75), antipsychotics (aOR: 1.70; 95% CI: 1.53–1.90), antiepileptic (aOR: 1.39; 95% CI: 1.22–1.57), and anti-Parkinson’s drugs (aOR: 1.52; 95% CI: 1.16–2.00) revealed the most significant associations (Figure 5).

Antihistamines, antiarrhythmics, antimuscarinic bronchodilators, skeletal muscle relaxants, and gastrointestinal antispasmodics did not show any noteworthy increases in risk factors in the study. Similarly, Richardson and colleagues [67] also discovered higher risks of developing dementia in individuals taking antidepressant, urological, and anti-Parkinson’s medications, while no link was found for gastrointestinal or antihistamine drugs. Stronger associations were also found in individuals diagnosed with VD before the age of 80 rather than AD, suggesting that the mechanism of anticholinergic action might affect vascular and inflammatory pathways along with inhibiting cholinergic actions [70]. Zheng and coworkers pooled fourteen longitudinal and case–control studies of 1,564,181 participants. They concluded that the use of anticholinergics was linked to a higher risk (1.2 folds) of developing ACD and AD [71]. In a population-based study (*n* = 750; age > 65), individuals taking anticholinergics had a higher probability of cognitive impairment in comparison to non-users (OR: 3.18; 95% CI: 1.93–5.23; *p* < 0.001) [72]. A cohort study in three French cities revealed a 1.4 to 2.0-fold dementia risk in anticholinergic users in comparison to the non-users [66]. The increased risks of dementia were linked to both low and high anticholinergic drug burdens (ACBs). The anticholinergics seemed to pose a greater risk of cognitive decline, which was accelerated with the changes in AD biomarkers [73]. Taylor-Rowan et al. assessed the risk factor of high anticholinergic burden in elders with no known cognitive issues, who may have experienced a significant rise in their risk of cognitive decline or dementia, potentially up to 227% [74]. Compared to other antidepressants, paroxetine was associated with the highest risk of dementia and AD [75]. Among the anticholinergic drugs, the dementia risk was pronounced with the usage of anti-Parkinson’s, urological, and antidepressant medications [63,71,76,77]; yet, cardiovascular and gastrointestinal medications may offer some level of protection [71]. Additionally, the risk of dementia and AD was directly proportional to the dosage of anticholinergics used (1.6, 2.1, 2.6, and 2.6 times increased risk with one, two, three, and four anticholinergic drugs) [78]. Among the antidepressants, clomipramine, trimipramine, imipramine, amitriptyline, doxepin maprotiline, and tranylcypromine were linked to a higher likelihood of developing dementia in a 12-year follow-up study [79]. Anticholinergic drugs do not just hinder cognitive function by reducing ACh neurotransmission, they also speed up neurodegeneration by inhibiting an ACh-dependent anti-inflammatory system [80].

On the contrary, no significant association between anticholinergics and dementia risk (HR: 1.043; 95% CI: 0.958–1.212, *p*: 0.139) was observed in a nationwide 15-year follow-up study in Taiwan. However, male patients, age group 65 to over 80 years, long anticholinergic treatment (≥4 years), and high ACB were associated with dementia risk [81]. Low et al. found no significant effect of anticholinergics on cognitive decline in persons in their early to mid-60s [82]. There was no significant difference in the likelihood of developing dementia between users and non-users during an 8-year monitoring period in a French cohort study [65]. The beta-blocker therapy also did not show a higher chance of developing ACD, AD, or mixed dementia (HR: 1.15; 95% CI: 0.80–1.66; *p*: 0.44; HR: 0.85; 95% CI: 0.48–1.54; *p*: 0.59 and HR: 1.35; 95% CI: 0.56–3.27; *p*: 0.50, respectively), but was responsible for increased VD risks, irrespective of cardiovascular risk factors or history of coronary events, stroke, or heart failure [83]. Further research is required to investigate the mechanisms linking beta(β)-blockers to a higher VD risk. In a recent study, various subgroups of antihypertensive medication (AHM), including β-blockers, were associated with lower dementia risk in primary care patients [84]. Moreover, the permeability of β-blockers towards the BBB exerted neuroprotective activity by removing toxic aggregates through the glymphatic system [85]. Table 4 summarizes the results of the studies discussed above.

In short, no results supported the connection between an anticholinergic mechanism and an increased risk of dementia. Insufficient data could not confirm a causal mechanism for these drug categories. Nonetheless, in some cases, the advantages of anticholinergics may outweigh the disadvantages of specific clinical syndromes, supporting their suitable use for the elderly.

### 2.3. Benzodiazepine Drugs 

**Figure 6:**
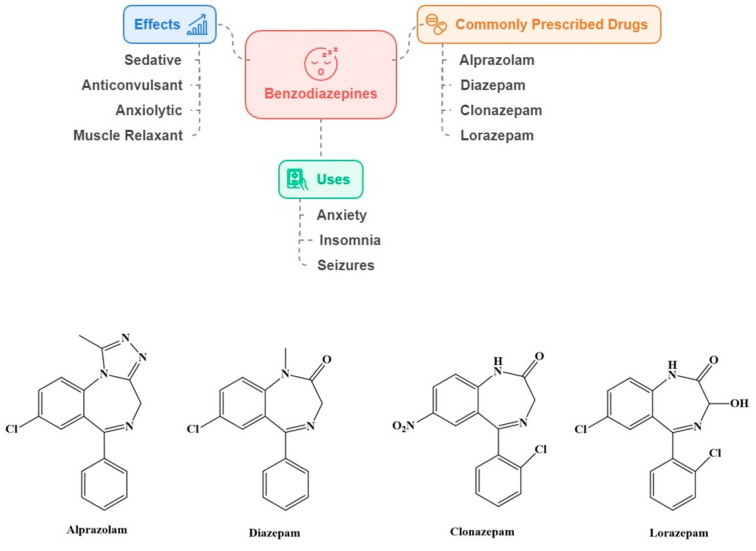
Common effects, uses, and examples of benzodiazepines.

Benzodiazepines (BZDs) are CNS depressants that bind to gamma (γ)-aminobutyric acid-A (GABA-A) receptors. These drugs increase the effect of GABA, resulting in sedative, anticonvulsant, anxiolytic, and muscle relaxant effects. Hence, they reduce the brain’s sensitivity to stimulation, leading to a calming effect. They were primarily utilized to address anxiety, insomnia, and seizures. The side effects of these medications include drowsiness, unsteadiness, and confusion. The most commonly prescribed drugs in this category include alprazolam, diazepam, clonazepam, and lorazepam (Figure 6).

#### Association of BZDs with Dementia and AD Risk

There has been a notable rise in AD and dementia risk among older individuals using BZDs with extended half-lives and for a prolonged period [86,87,88,89,90,91,92]. Additionally, AD patients prescribed with BZDs had a 41% increased risk of mortality [93] and a 50% increased risk of AD [91] in comparison to those who did not take these medications. Likewise, patients on hypnotic drugs had a 21% higher chance of developing dementia than those who do not use them (OR: 1.21, 95% CI: 1.13–1.29; *p* < 0.001) [94]. The combined results from a meta-analysis indicated a notable link between taking BZDs and the likelihood of developing dementia (OR: 1.38, 95% CI: 1.07–1.77). Moreover, no variation in the risk of dementia between individuals using short-acting and long-acting BZDs (RR: 1.09 vs. 1.24) was observed [95]. On the other hand, another study reported a higher risk of dementia in individuals using long-acting BZDs (OR: 1.21, 95% CI: 0.99–1.49) in comparison to those using short-acting BZDs (OR: 1.13, 95% CI: 1.02–1.26) [96].

There is a possibility that BZDs impact cognition and may raise the chance of AD by affecting hippocampal α5GABAA receptors [97]. Zhong and colleagues examined how long-term use of BZDs was linked to dementia by conducting a meta-analysis with 45,391 participants and 1891 cases of dementia. A possible link between an increase in BZD dose and a 22% higher risk of developing dementia (Risk ratio [RR]:1:22, 95% CI: 1.18–1.25) was reported in the study [98]. Mura et al. [99] observed that long-term BZD usage was linked to worse cognitive function, but was not associated with faster cognitive deterioration in older age. Pietrzak et al. [100] observed that elders with anxiety could be at a heightened risk of developing AD, which may lead to elevated levels of Aβ in individuals with MCI and AD. There was a significant increase in the risk of MCI among APOEe4 carriers (HR 1.30; 95% CI 1.14—1.50) [101].

However, some other studies disapprove of any correlation between BZD use and AD or cognitive loss [6,102,103,104,105]. Wu et al. found weak evidence to conclude any correlation between BZD use and dementia in an umbrella review comprising five meta-analyses and 30 observational studies [105]. The strength of evidence was weak. BZD use in patients did not show a link to dementia, with Adjusted risk ratios of 1.06 (95% CI: 1.02–1.10), 1.05 (95% CI: 1.01–1.09), and 1.05 (95% CI: 1.02–1.09) for low, medium, and high users of benzodiazepines [106]. Extended use of BZDs over 2 years was linked to higher chances of being diagnosed with dementia, yet the findings lack statistical significance at the 5% or 10% thresholds (1.190; 95% CI: 0.925–1.531 and 1.167; 95% CI: 0.919–1.483) [8]. As individuals grow older, the likelihood of sleep disorders rises, which can impact the brain’s ability to remove toxic substances like Aβ [107]. Hence, it can be suggested that BZDs might indirectly have a protective impact on preventing AD by enhancing sleep quality [108]. Moreover, BZDs protect against glutamate excitotoxicity in AD [109]. Table 5 summarizes the results of the studies discussed above.

The data compiled in this review do not warranty increased dementia/AD risk with BZD use. The use of BZDs had both advantages and disadvantages. Healthcare providers need to be aware of the dangers of long-term benzodiazepine use, such as dependence, withdrawal, and cognitive impairment, to promote safe and effective medication use. The injudicious use of medication can be prevented by alternate medications such as cognitive behavioral therapy for insomnia (CBT-i); the use of melatonin could also be a safer choice as medication, but further evidence would be needed to evaluate its long-term effect on health. Treating anxiety in elderly patients may be better addressed using alternative medications such as serotonin uptake inhibitors like sertraline or other compounds.

### 2.4. Proton Pump Inhibitors (PPIs)

**Figure 7:**
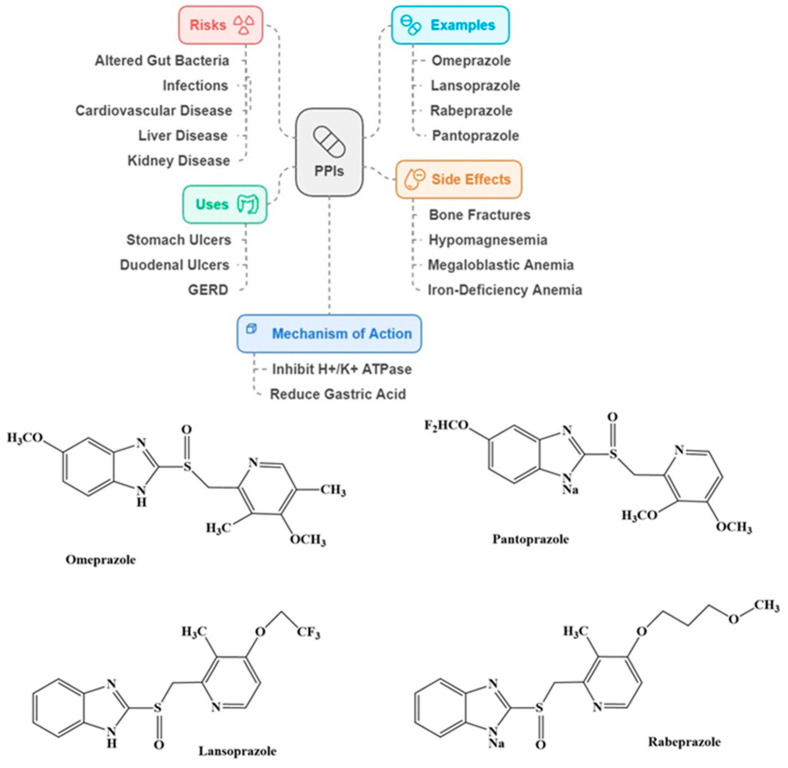
Summary and structures of some common proton pump inhibitors.

PPIs rank high among the drugs that are frequently prescribed on a global scale with an expected expenditure of USD 9 billion worldwide [111]. These medications inhibit the hydrogen-potassium adenosine triphosphatase enzyme (H^+^/K^+^ ATPase; Proton pump) present in the cells lining the stomach that produce acid. They are mainly used to treat stomach/duodenal ulcers and gastroesophageal reflux disease (GERD), as they assist in healing ulcers and reducing acid reflux by reducing gastric acid production. Some of the commonly prescribed PPIs include omeprazole, lansoprazole, rabeprazole, and pantoprazole (Figure 7). Although they work well for treating specific gastrointestinal problems, the potential risks require close supervision and evaluation of discontinuation methods to guarantee the best health results for the elderly. Therefore, it is advised to regularly assess PPI treatment to confirm that the lowest effective dose is being utilized for symptom management. Extended use of PPIs can alter the gut bacteria equilibrium, leading to a higher chance of infection, particularly pneumonia and *Clostridium difficile* (CDI). They have also been associated with a risk of cardiovascular, liver, and kidney disease [112]. PPIs hinder the absorption of vitamins and minerals like vitamin B12, magnesium, calcium, and iron, causing bone fractures, hypomagnesemia, and megaloblastic and iron-deficiency anemia [110]. PPTs can cross the BBB [113], and have also been reported to block acetylcholine synthesis by *Choline acetyltransferase* (ChAT), thereby potentially disrupting neurotransmission [114].

#### Association of PPIs with Dementia and AD Risk

**Figure 8:**
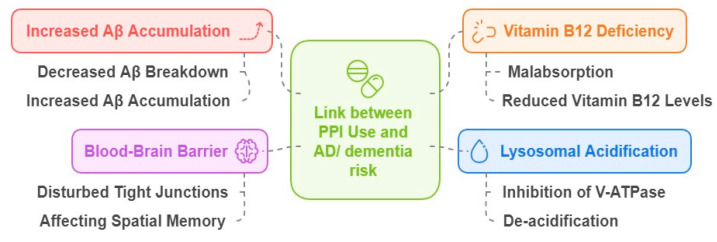
Proton pump inhibitor pathways linked to AD risk.

In recent years, some studies proposed a possible link between PPI use and dementia risk [9,115,116,117]. A 51% increased risk of developing dementia was observed by Welu et al. [118] in war veterans. A significant increase in dementia risk was also observed in Asian [119] and German [120] populations. A two-fold increased risk of developing dementia by PPI users [117] raised safety concerns over the use of PPIs. The association between PPIs and dementia may be due to increased Aβ accumulation that is linked to dementia, or by PPI-induced malabsorption, leading to vitamin B12 deficiency. Considerably reduced levels of vitamin B12 were reported in dementia and AD patients in comparison to the healthy controls [121,122,123]. Acidification of lysosomes was important for activated microglia to degrade Aβ [124] and was seen as an important factor in AD pathogenesis. PPIs are lipophilic weak bases that can cross the BBB and block lysosomal vacuolar (V)-ATPase, inhibiting lysosomal acidification. Eventually, the de-acidification results in a decrease in Aβ breakdown, and an increase in Aβ accumulation. PPIs increase their uptake by disturbing tight junctions in the BBB, affecting spatial memory [125,126] (Figure 8).

Some other studies [127,128,129,130] found no association between the risk of dementia or AD and the use of PPIs, including in individuals with significant long-term exposure [131]. An increased dosage of PPIs did not show a higher risk for either AD or non-AD dementias (OR: 1.20; 95% CI: 0.91–1.61 and OR: 0.95; 95% CI: 0.74–1.22, respectively) [132]. Another meta-analysis study including 642,305 participants found no significant link between PPI use and dementia (HR: 1.04; 95% CI: 0.92–1.15) [10]. No connection between PPI and dementia risk was seen in the extensive meta-analysis (HR: 0.98; 95% CI: 0.85–1.13) either [133]. A clinical trial also supported the above results, where no statistically significant association (OR: 1.20; 95% CI: 0.81–1.78) between dementia and PPI (pantoprazole, 40 mg) use was seen [134]. A comprehensive review published last year, including meta-analyses and experimental and observational studies, also found no strong link between PPI use and the onset of cognitive decline or dementia [135]. Table 6 summarizes the results of the studies discussed above.

While looking for further conclusive proof, doctors need to prescribe PPIs by weighing the advantages against possible drawbacks. The legitimate use of PPI medications should not be denied due to worries about dementia risk. Increased awareness and following evidence-based recommendations are crucial for maximizing PPI treatment and minimizing associated risks. Regularly checking and carefully planning how to reduce PPI prescriptions are important for effective management. For those with suitable reasons for PPI treatment, it is important to always start with the smallest effective dose and avoid unnecessary long-term or continuous treatment in appropriate individuals. A gradual reduction in dosage may be suitable for individuals who no longer have symptoms, and/or need to discontinue the PPI. Regular monitoring of serum vitamin B12 is necessary, particularly in older patients on long-term PPI treatment. Using cyanocobalamin may enhance vitamin B12 levels in cases where discontinuing PPI therapy is not possible.

### 2.5. Statins

**Figure 9:**
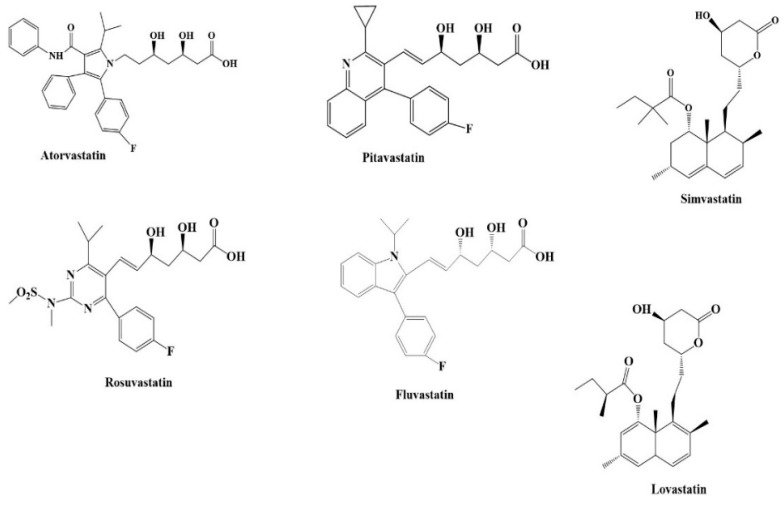
Structures of some statins.

Statins are utilized in the treatment of high cholesterol levels and the management of atherosclerotic cardiovascular disease (CVD). Statins inhibit the enzyme hydroxymethylglutaryl-CoA (HMG-CoA) reductase in the cholesterol biosynthesis pathway. Statins reduce total cholesterol levels, low-density lipoprotein (LDL), and triglycerides (TGs). Examples of FDA-approved statins are atorvastatin, rosuvastatin, simvastatin, pravastatin, fluvastatin, lovastatin, and pitavastatin (Figure 9). The side effects of statins include fatigue, myopathy, and hepatic injury [137].

#### Association of Statins with Dementia and AD Risk

**Figure 10:**
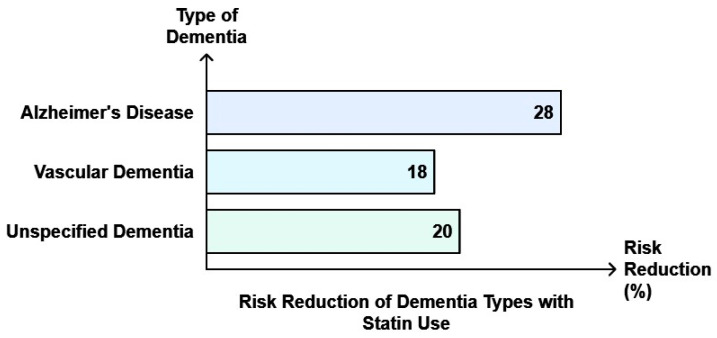
Statin use reduces dementia and AD risk. The graph was plotted using data reported by Ren et al., 2024 [156].

In 2012, the US FDA expressed worries over the negative cognitive impact of statins [138], after some studies indicated their effect on memory [136]. The decreased statin use following the negative publicity resulted in increased myocardial infarction and CVD mortality [139]. Later, several other studies found that statin use does not increase dementia or AD risk [140,141,142,143,144], but helps reduce them instead. Studies suggested the role of statins in decreasing proinflammatory markers and controlling microglial activation in NDs [145] and by modulating G protein-gated inwardly rectifying potassium (GIRK) channel function [146] in neurons. Jeong and coworkers [147] found that less persistent use of statins (<540 days) was associated with increased AD risk, while consistent use of statins decreased the risk of AD [Adjusted hazard ratio (aHR); 95% CI  =  0.87 (0.80–0.95)]. A meta-analysis with 30 observational studies (*n* = 9,162,509) indicated that statin use was associated with reduced dementia risk (Risk ratio [RR]: 0.83; 95% CI: 0.79–0.87), AD (RR: 0.69; 95% CI: 0.60–0.80, *p* < 0.0001), and VD (RR: 0.93; 95% CI: 0.74–1.16, *p* = 0.54) [148]. No adverse effect of statins was observed in randomized clinical trials and observational studies constituting 1,404,459 participants [149]. During follow-up, individuals taking statins had a lower chance of developing dementia, or cognitive impairment without dementia [150], and AD [151]. Similar results were reported by a nationwide cohort study in Taiwan where statin use significantly lowered the dementia risk in the aging population [152]. The outcomes were alike for lipophilic and hydrophilic statins and were additionally more pronounced for potent statins than the less potent statins [153]. Statins may play a therapeutic role in preventing Aβ-induced toxicity and improving insulin signaling by activating AMP-activated protein kinase (AMPK) [154] and decreasing neuroinflammation [155]. Statin use was linked to a reduced risk of AD by 28%, VD by 18%, and unspecified dementia by 20% [156] (Figure 10).

With the rise in life expectancy, it will be crucial to define precise guidelines for prescribing statin therapy to elderly individuals to promote a healthier aging society. It was noted that several factors must be considered when utilizing statin therapy in elderly patients, including multiple health conditions and the use of polymedications, which can impact the effectiveness and safety of treatment. Drug interactions frequently occur in patients who are taking several medications. Lovastatin, simvastatin, and atorvastatin, metabolized by cytochrome p450, may interact with drugs like diltiazem, verapamil, amiodarone, and azole antifungals. These interactions were common in patients with atherosclerotic cardiovascular disease (ASCVD). In the elderly, pravastatin and rosuvastatin were preferable due to their lower risk of drug interactions and improved safety record [157]. On the other hand, discontinuing the treatment led to a higher likelihood of cardiovascular issues and increased risk of death [158]. Table 7 summarizes some of the important results discussed above.

## 3. Methods

A comprehensive search was done for the published scientific research available on various databases (PubMed, Google Scholar, and Web of Science) until September 2024. These included observational studies, randomized case–control studies, meta-analysis and systematic reviews, and clinical studies on medications associated with AD and dementia risk. The search terms used were “drugs” and “medication” with the filters “Alzheimer’s disease risk” and “dementia risk”. Exclusion criteria: articles not specifying AD, or dementia were excluded. Non-English literature was excluded.

## 4. Conclusions

Several searches using various factors and databases were carried out to find a piece of concrete evidence between the use of several medications and increased risks of dementia or AD, yet the results were inconclusive. The conflicting results in research could be due to the variability in the study design (sample size, ethnicity, methodology); biological differences (pharmacokinetic, pharmacodynamic, multiple health conditions); and other cofounding variables (sociodemographic factors, polypharmacy, comorbidities). Moreover, the studies considered only prescription medicine; therefore, it was impossible to determine if patients took additional over-the-counter drugs (allergy medicine, motion sickness patches, cough syrups, muscle relaxants). Like other geriatric syndromes, the risk factors for cognitive impairment are complex and could vary among individuals. Cognitive impairment is largely influenced by biological factors such as age, gender, and genetics. Furthermore, cognitive health can be significantly affected by psychological factors like depression and anxiety. The databases were expected to be trustworthy, but the accuracy of the recorded information could be ambiguous at times. With this information and the possibility of confusion, it is extremely difficult to determine the specific amount of risk linked to any category or specific medication.

In the elderly, polypharmacy might result in potential drug–drug interaction; therefore, the patients should be regularly evaluated for their medications and any negative effects, opting for single-drug therapy over fixed-dose combinations, selecting medications with confirmed effectiveness, and discontinuing drugs as early as feasible. Adjusting the dosage timing for elderly patients susceptible to nutrient deficiencies can also help lessen interactions and side effects caused by multiple medications used by this population. In 2019, the American Geriatrics Society (AGS) Beers Criteria advised against using drugs with potent anticholinergic properties in elderly individuals with dementia or cognitive decline [160]. Instead, medications with a lower ACB like SSRIs (selective serotonin reuptake inhibitors) and SNRIs (serotonin and norepinephrine reuptake inhibitors) were seen as safer options. Several other criteria limited the PPI usage for not more than 8 weeks in the elderly [159,161]. Other risk factors for cognitive decline need to be acknowledged and possibly addressed, along with a focus on non-pharmacological treatments. Hence, it is crucial to consider the pros and cons of every medication. The possibility exists that the danger of dementia could be significantly lower than the danger to one’s health of not using the medication as directed. Hence, individuals should refrain from discontinuing prescribed medications without consulting their doctor.

## Figures and Tables

**Table 1:** A generalized view on medication use in adult (<60 years) and elderly (60+) groups.

Medication Type	Young Adults (%)	Elderly (%)	Ref.
Analgesics	20–60	70–85	[11,12,13,14]
Anticholinergics	20–25	30–50	[15,16]
Benzodiazepines	5–10	30–40	[17,18]
Proton pump inhibitors	2–5	35–50	[19]
Statins	15–25	35–60	[20,21,22]

**Table 2:** Drugs, their targets, protein expression, and location of these targets in different cells and tissues.

Drug	Target	^1^ Protein Expression and Location	^1^ Tissue RNA Expression	^1^ Cell-Type RNA Expression
Analgesics	Cox 1 and 2, opioid receptors (μκ Others (BACE1, PPARγ)	Cox1: Cytoplasmic expression at variable levels in several tissues, high expression in squamous epithelia, megakaryocytes, fallopian tube, brain, and subsets of cells in tissue stroma. Localized to the Golgi apparatus, Vesicles	Tissue-enhanced (Intestine, Skin, Urinary bladder). Skin—Cornification (mainly). Low human brain regional specificity. Macrophages and Microglia—Immune response (mainly)	Group-enriched (granulocytes, Glandular and luminal cells). Cell-type-enriched (Adrenal gland—Macrophages, Heart muscle—Fibroblasts, Skeletal muscle—Fibroblasts, Skin—Keratinocyte (other), Spleen—Platelets)
		Cox 2: Cytoplasmic and membranous expression in selected tissues, including seminal vesicle, urinary bladder, and gall bladder. Localized to the Vesicles, Cytosol	Tissue-enhanced (Bone marrow, Seminal vesicle, Urinary bladder). Tissue-enhanced (Bone marrow, Seminal vesicle, Urinary bladder). Low human brain regional specificity. Neurons—Mixed function (mainly)	Group-enriched (Basal prostatic cells, granulocytes, Langerhans cells, monocytes, Macrophages, Alveolar cells type 1). Cell-type-enriched (Heart muscle—Fibroblasts)
		Opioid receptor μ: Membranous expression in seminiferous tubules. Soma, dendrite, axon, and synapse in neurons.	Tissue-enhanced (Brain, Testis). Low human brain regional specificity. Sub-cortical—Mixed function (mainly)	Cell-type-enhanced (Excitatory neurons, Early spermatids, Late spermatids, Inhibitory neurons, Microglial cells). Cell-type-enriched (Adrenal gland—Adrenal medulla cells, Testis—Early spermatids, Testis—Late spermatids)
		Opioid receptor κ: Cytoplasmic expression in CNS. Localized to the Plasma membrane, Nucleoplasm, Cytosol	Tissue-enhanced (Brain, Prostate, Skeletal muscle), Low human brain regional specificity, Neurons—Mixed function (mainly)	Cell-type-enhanced (Prostatic glandular cells, Inhibitory neurons, Extravillous trophoblasts, Glandular and luminal cells, Excitatory neurons, Leydig cells). Cell-type-enriched (Colon—Colon enteroendocrine cells, Minor Salivary Gland—Adipocytes (Minor salivary gland), Prostate—Prostate glandular cells)
		BACE1: Granular cytoplasmic expression in several tissues. Localized to the Plasma membrane	Group-enriched (Brain, Pancreas). Low human brain regional specificity. White matter—Myelination (mainly)	Cell-type-enhanced (Late spermatids, Cone photoreceptor cells, Oligodendrocytes)
		PPARγ: Cytoplasmic and nuclear expression in several tissues. Localized to the Nucleoplasm, Vesicles	Tissue-enhanced (Adipose tissue). Low human brain regional specificity. Neurons—Mixed function (mainly)	Cell-type-enhanced (Extravillous trophoblasts, Adipocytes, Distal enterocytes, Cytotrophoblasts, Syncytiotrophoblasts)
Anticholinergics	mAChR (M1–M5)	M1: Cytoplasmic expression in pyramidal neurons and Purkinje cells. Soma, dendrite, and synapse in neurons.	Group-enriched (Brain, Prostate, Salivary gland), Group-enriched (Amygdala, Basal ganglia, Cerebral cortex, Hippocampal formation, White matter), Neurons—Mixed function (mainly)	Cell-type-enriched (Prostatic glandular cells), Cell-type-enriched (Heart muscle—Cardiomyocytes, Liver—Hepatic stellate cells, Testis—Early spermatids, Testis—Late spermatids), Group-enriched (Brain, Prostate, Salivary gland)
		M2: Ubiquitous cytoplasmic expression in all tissues at variable levels. Localized to the Plasma membrane, Nucleoli, Golgi apparatus, Primary cilium, Basal body	Tissue-enhanced (Heart muscle, Intestine), Heart muscle—Heart development (mainly), Low human brain regional specificity, Hindbrain—Mixed function (mainly)	Cell-type-enriched (Heart muscle—Cardiomyocytes, Liver—Hepatic stellate cells, Testis—Early spermatids, Testis—Late spermatids) Group-enriched (Inhibitory neurons, Cardiomyocytes, Excitatory neurons)
		M3: General cytoplasmic expression at variable levels. Localized to the Plasma membrane	Tissue-enhanced (Salivary gland), Salivary gland—Salivary secretion (mainly) Low human brain regional specificity, Astrocytes—Astrocyte-neuron interactions (mainly)	Cell-type-enriched (Heart muscle—Smooth muscle cells, Minor Salivary Gland—Minor salivary glandular cells, Skin—Eccrine sweat gland cells, Thyroid gland—Endothelial cells), Group-enriched (Excitatory neurons, Inhibitory neurons)
		M4: Cytoplasmic and membranous expression in several tissues, including the brain and intestines Localized to the Golgi apparatus and Nucleoplasm	Tissue-enhanced (Brain, Intestine, Lymphoid tissue), Human brain regional-enhanced (Basal ganglia), Neurons—Mixed function (mainly)	Cell-type-enriched (Colon—Colon enteroendocrine cells, Skin—Keratinocyte (granular), Testis—Early spermatids, Testis—Late spermatids)
		M5: Tissue profile NA. Membrane, Intracellular (different isoforms)	Tissue-enriched (Brain), Low human brain regional specificity, Oligodendrocytes—Mixed function (mainly)	Cell-type-enriched (Oligodendrocytes, Testis—Early spermatids, Testis—Late spermatids)
Benzodiazepines	GABA-A receptor	Selective expression in neuropil and subset of neurons, Localized to the Plasma membrane, Nucleoplasm, Synapse in neurons	Group-enriched (Brain, Retina) Brain—Synaptic signal transduction (mainly) Low human brain regional specificity Neurons—Mixed function (mainly)	Cell-type-enriched (Adrenal gland—Adrenal medulla cells, Stomach—Gastric enteroendocrine cells, Testis—Early spermatids, Testis—Late spermatids)
Proton pump inhibitors	H/K-ATPase	Membranous expression in most tissues. Localized to the Vesicles, Plasma membrane	Tissue-enhanced (Parathyroid gland), Parathyroid gland—Vesicular transport (mainly). Low human brain regional specificity, Neurons and Synapses—Synaptic function (mainly)	Cell-type-enriched (Adrenal gland—Adrenal cortex cells, Lung—Alveolar cells type 2, Thyroid gland—Thyroid glandular cells)
Statins	HMG-CoA reductase	Ubiquitous cytoplasmic expression. Membrane, Intracellular (different isoforms)	Tissue-enhanced (Liver) Liver and Intestine—Lipid metabolism (mainly); Low human brain regional specificity. Neurons—Mixed function	Cell-type-enriched (Adrenal gland—Adrenal cortex cells, Lung—Alveolar cells type 2, Minor Salivary Gland—Minor salivary gland basal cells)

BACE: β-secretase; COX: cyclooxygenase; GABA: γ-aminobutyric acid; H/K-ATPase: hydrogen-potassium adenosine triphosphatase; HMG CoA reductase: 3-hydroxy-3-methylglutaryl coenzyme A; mAChR: muscarinic acetylcholine receptors; PPARγ: peroxisome proliferator-activated receptor gamma. ^1^ The Human Protein Atlas.

**Table 3:** Association between use of analgesics and AD/dementia risk.

Study	Population Sample	Analgesic Type	AD/Dementia Risk Factor	Statistics	Ref.
Prospective Study—UK Biobank	*n* = 194,758 (aged ≥ 60)	Aspirin, Paracetamol, 2–3 NSAIDs	Frequent use of paracetamol, as opposed to ibuprofen, was linked to a notably increased likelihood of developing dementia in elderly	Aspirin HR: 1.12 (CI: 1.01–1.24, *p* < 0.05) Paracetamol HR: 1.15 (CI:1.05–1.27, *p* < 0.01) 2–3 NSAIDs HR: 1.2 (CI:1.08–1.33, *p* < 0.05)	[35]
Prospective national cohort study—Israel	*n* = 91,307 (aged ≥ 60)	Opioids	Linked with an increased dementia risk	aHR = 1.39, 95% CI = 1.01–1.92, *p* < 0.05 in opioid exposed group aged 75+ yrs	[41]
Community-based cohort—West King County, US	*n* = 3392 (aged ≥ 65)	NSAIDs	Heavy NSAID users had increased incidence of dementia and AD	Dementia: aHR 1.66 95% CI: 1.24–2.24 AD: aHR:1.57 95% CI: 1.10–2.23	[44]
Population Cohort Study- Korean patients	*n* = 1,261,682	Opioids	Increased AD and dementia risk	Dementia: 15%; AD: 15%; Unspecified Dementia: 16%	[40]
Longitudinal Study—Baltimore	*n* = 1686	NSAIDs	No association was found between AD risk and use of acetaminophen AD risk decreased with increasing duration of NSAID use	RR = 1.35; 95% CI: 0.79–2.30 2 or more years NSAID use, RR:0.40 95% CI: 0.19–0.84; less than 2 years RR: 0.65 95% CI: 0.33–1.29	[50]
Population-based autopsy cohort—ACT Study	*n* = 257	NSAIDs	Increased dementia risk	1000–2000 SDD [RR] 2.16, 95% [CI] 1.02–4.25; >2000 SDD [aRR] 2.37, 95% CI 1.24–4.67	[45]
Nested Case–Control Study UK Biobank	*n* = 500,000 (age 40–69 yrs)	Opioids	Increased dementia risk Risk increased with more opioid prescriptions	OR (1–5 prescriptions): 1.21 (CI: 1.07–1.37, *p*: 0.003); OR (6–20 prescriptions): 1.27 (CI: 1.08–1.50, *p*: 0.003); OR (>20 prescriptions): 1.43 (CI: 1.23–1.67, *p* < 0.001)	[43]
Community-based autopsy cohort—ACT Study	*n* = 420	Opioids NSAIDs	The use of prescription opioids does not have a connection with dementia-related neuropathological alterations, although frequent use of NSAIDs may be linked	For neuritic plaques, the aRR [95% CI] was 0.99 [0.64–1.47] for 91+ TSDDs of opioids; heavy NSAID use had a higher risk of neuritic plaques (RR 1.39 [1.01–1.89])	[46]
Systematic Review and Meta-Analysis (6 cohort; 3 case–controls)	*n* = 13,211 (cohorts) *n* = 1443 (case–controls) (age > 55 yrs)	NSAIDs	Decreased AD risk	Pooled RR for AD:0.72 95% CI: 0.56–0.94 (<1 month) RR: 0.95 CI: 0.70–1.29; (<24 months) RR:0.83 CI: 0.65–1.06; (>24 months) RR: 0.27 CI: 0.13–0.58	[52]
Systematic Review and Meta-Analysis (17 epidemiological studies from 8 countries—US, Australia, Canada, China, Finland, Italy, Netherlands, UK)		NSAIDs	Decreased AD risk	Combined OR: 0.55, *p* < 0.0001	[49]
A Finnish nationwide nested case–control study MEDALZ	*n* = 70,718 (mean age 80 yrs)	Opioids	No increased AD risk even for longer duration or higher dose	aOR: 1.00 95% CI: 0.98–1.03 cumulative use for >365 days: aOR = 1.02, 95% CI = 0.96–1.08 >90 TSDs: aOR = 1.02, 95% CI = 0.98–1.07	[56]
Meta-analysis (16 cohort studies)	*n* = 236,022	NSAIDs	Reduced AD risk	Global RR: 0.81 CI: 0.70–0.94; Europe: RR: 0.72; Asia: no association	[57]
Prospective Cohort Study-Northwest US	*n* = 3434 (median age 74 yrs)	NSAIDs Opioids	Individuals who had high levels of opioid or NSAID consumption were found to have a slightly elevated risk of dementia compared to those with minimal or no usage	Cumulative opioid use: HRs for dementia: 1.06 95% CI = 0.88–1.26 for 11 to 30 TSDs; HR: 0.88 (95% CI = 0.70–1.09) for 31 to 90 TSDs; and HR:1.29 (95% CI = 1.02–1.62) for 91 or more TSDs. A similar pattern was seen for NSAID use	[55]
Clinical trial double-blind, placebo-controlled study—Northwest Phoenix metropolitan area	*n* = 44	NSAID (indomethacin)	Protective against cognitive decline in mild to moderately impaired AD	Improved cognitive tests *p* < 0.003	[51]
Prospective, population-based cohort study—Rotterdam, Netherlands	*n* = 6989 (age ≥ 50)	NSAIDs	No association with a reduction in the risk of VD	AD: RR: 0.95 95% CI: 0.70–1.29 (short-term use); RR: 0.83 95% CI: 0.62–1.11 (intermediate-term use); and RR:0.20 95% CI: 0.05–0.83 (long-term use)	[58]

AD: Alzheimer’s disease; aHR: Adjusted hazard ratio; aOR: Adjusted odds ratio; aRR: Adjusted risk ratio; CI: confidence interval; HR: Hazard ratio; NSAIDs: Non-Steroidal Anti-Inflammatory Drugs; RR: Risk ratio; OR: Odds ratio; SDDs: Standard daily doses; TSD: Total standardized daily dose; VD: Vascular dementia.

**Table 4:** Association between anticholinergics use and AD/dementia risk.

Study Type	Population Sample	Effect on Dementia/AD Risk	Statistical Data	Ref.
German Cohort Study	*n* = 2605, (age > 75 ± 4.5 yrs)	Increased risk of dementia	Dementia: HR: 2.08; AD: HR: 1.63	[66]
UK Case–control study	*n* = 40,770 (age ≥ 65 yrs)	Dementia was associated with an increasing average ACB score	aOR for any anticholinergic drug with an ACB score of 3 was 1.11 (95% confidence interval 1.08 to 1.14)	[67]
Population-based Cohort Study— South Korea	*n* = 550,000 (age ≥ 60 yrs)	AD risk was higher in subjects with an increased number of prescriptions	HR (95% confidence interval (95% CI)) 0.99 (0.95–1.03), 1.19 (1.12–1.26), 1.39 (1.30–1.50); in the 10–49 doses/year, 50–119 doses/year, and ≥120 doses/year groups HR higher in the young-old subgroup (60–64 years old) [HR (95% CI) 1.11 (1.04–1.22), 1.43 (1.25–1.65), 1.83 (1.56–2.14); in the 10–49 doses/year, 50–119 doses/year, and ≥120 doses/year groups	[68]
Nested Case–Control Study—UK	*n* = 284,343	Higher dementia risk	Antidepressants: aOR, 1.29; 95% CI, 1.24–1.34, Anti-Parkinson’s drugs: aOR, 1.52; 95% CI, 1.16–2.00, Antipsychotics: aOR, 1.70; 95% CI, 1.53–1.90, Bladder antimuscarinic drugs: aOR, 1.65; 95% CI, 1.56–1.75, and Antiepileptic drugs: aOR, 1.39; 95% CI, 1.22–1.57 all for more than 1095 TSDDs	[70]
Population-based cohort recruited from 3 French cities	*n* = 6912 (age > 65 yrs)	Increased risk of cognitive decline and dementia	HR: 1.65; 95% CI, 1.00–2.73	[65]
Systematic Review and Meta-Analysis (France, UK, Germany, US, Nigeria, Turkey, Netherlands, China, Taiwan)	*n* = 1,564,18	Increased risk of ACD and AD (anti-Parkinson’s, urological, antidepressants) Negative association with dementia (anticholinergic cardiovascular and gastrointestinal drugs) No association (antipsychotic, analgesic, and respiratory anticholinergic)	Anti-Parkinson’s (RR = 1.39, 95% CI: 1.26–1.53, I^2^ = 0, 95% CI: 0–90%, *p* = 0.504), Urological drugs (RR = 1.27, 95% CI: 1.12–1.44, I^2^ = 94.4%, 95% CI: 87%–98%, *p* < 0.001), and Antidepressants (RR = 1.19, 95% CI: 1.15–1.22, I^2^ = 52.6%, 95% CI: 0–83%, *p* = 0.077). Anticholinergic cardiovascular (RR = 0.97, 95% CI: 0.95−0.996, I^2^ = 0, 95% CI: 0–90%, *p* = 0.721) Gastrointestinal (RR = 0.95, 95% CI: 0.91−0.99, I^2^ = 48.1%, 95% CI: 0–85%, *p* = 0.146)	[72]
Population-Based Study— Italy	*n* = 750, (age > 65 yrs)	Cognitive impairment	OR: 3.18 95% CI: 1.93–5.23, *p* < 0.001	[73]
ADNI cohort	*n* = 688 (mean age 73.5 yrs)	Increased dementia risk	HR:1.47, *p* = 0.02	[75]
Prospective and retrospective longitudinal cohort and case–control observational studies	*n* = 968,428 (age ≥ 65 yrs)	Increased risk of cognitive decline or dementia	OR 1.47, 95% CI 1.09 to 1.96	[76]
Longitudinal Study—England and Wales	*n* = 13,004 (age ≥ 65 yrs)	Increased cumulative risk of cognitive impairment and mortality	A decline in MMSE score (95% confidence interval (CI) = 0.03–0.64, *p* = 0.03)	[61]
A Nationwide 15-Year Follow-Up Cohort Study—Taiwan	*n* = 790,240 (age > 65 yrs)	No significant association	HR: 1.043 (95% CI: 0.958–1.212, *p* = 0.139)	[83]
Retrospective cohort study used the Humana Research Database	*n* = 12,209	Increase AD and dementia risk	1.6 (95% CI 1.4–1.9), 2.1 (95% CI 1.7–2.8), 2.6 (95% CI 1.5–4.4), and 2.6 (95% CI 1.1–6.3) times increased risk	[80]
UK Biobank Cohort study	*n* = 171,775	Increase dementia risk	HR: 1.094; 95% CI: 1.068–1.119	[79]
Prospective cohort study conducted within Kaiser Permanente Washington (KPWA)	*n* = 3059	Increase AD and dementia risk	0–90 TSDDs: HR = 1.69, 95% CI = 1.18–2.42; 91–365 TSDDs: HR = 1.40, 95% CI = 0.88–2.23; 366–1095 TSDDs: HR = 2.13, 95% CI = 1.32–3.43; ≥1095 TSDDs: HR = 1.42, 95% CI = 0.82–2.46	[77]
Nationwide 12-year cohort study—Taiwan	*n* = 16,412 (age > 50 yrs)	Bladder anticholinergics increased dementia risk	Dementia aHR 1.15 (95% CI = 0.97–1.37) in the 85–336 cDDD group, and 1.40 (95% CI = 1.12–1.75) in the ≥337 cDDD group	[78]
Randomly selected community-based	*n* = 2058 (age 60–64 yrs)	Anticholinergic medication is associated with lower level of complex attention in the young-old, but not with greater cognitive decline over time		[84]
Prospective cohort study—Seattle, Washington	*n* = 3434 (age ≥ 65 yrs)	Increased risk for dementia	aHR: 0.92 (95% CI, 0.74–1.16) for TSDDs of 1 to 90; aHR:1.19 (95% CI, 0.94–1.51) for TSDDs of 91 to 365; aHR:1.23 (95% CI, 0.94–1.62) for TSDDs of 366 to 1095; and aHR: 1.54 (95% CI, 1.21–1.96) for TSDDs greater than 1095. A similar pattern for AD	[63]
Longitudinal cohort study—Montpellier region of southern France	*n* = 372 (age > 60 yrs)	Significant cognitive functioning impairments and high chances of being categorized as MCI impaired, with no higher risk of dementia	OR: 5.12, *p* = 0.001	[64]
Population-based prospective study—Malmo, Sweden	*n* = 18,063 (mean age 68.2 yrs)	β-blockers not associated with increased risk ACD, AD, and mixed dementia	ACD:HR:1.15; 95% CI 0.80–1.66; *p* = 0.44; AD:HR:0.85; 95%CI 0.48–1.54; *p* = 0.59 and Mixed dementia: HR:1.35; 95%CI 0.56–3.27; *p* = 0.50	[86]
Longitudinal cohort study—Netherlands	*n* = 133,355	AHM, ARBs, CCBs, and Ang-II-stimulating AHM were associated with lower dementia risk	ARBs [HR = 0.86 (95% CI = 0.80–0.92)], β-blockers [HR = 0.81 (95% CI = 0.75–0.87)], CCBs [HR = 0.77 (95% CI = 0.71–0.84)], and diuretics [HR = 0.65 (95% CI = 0.61–0.70)] were associated with significantly lower dementia risks; β-blockers [HR = 1.21 (95% CI = 1.15–1.27)] and diuretics [HR = 1.69 (95% CI = 1.60–1.78)] were associated with higher, CCBs with similar, and ARBs with lower [HR = 0.83 (95% CI = 0.80–0.87)] mortality risk. Dementia [HR = 0.88 (95% CI = 0.82–0.95)] and mortality risk [HR = 0.86 (95% CI = 0.82–0.91)] were lower for Ang-II-stimulating drugs	[87]
Danish Population-based study	*n* = 69,081 (median age 64.4 yrs)	Highly permeable β-blockers protect against AD by promoting waste brain metabolite clearance.	−0.45%, *p* < 0.036	[88]

AD: Alzheimer’s disease; ACB: Anticholinergic cognitive burden; aHR: Adjusted hazard ratio; aOR: Adjusted odds ratio; ACD: All-cause dementia; cDDD: Cumulative defined daily dose; AHM: Antihypertensive medication; ANG-II: Angiotensin-II-receptor; ARBs: Angiotensin receptor blockers; CCBs: Calcium channel blockers; CI: confidence interval; HR: Hazard ratio; RR: Risk ratio; MCI: Mild cognitive decline; OR: Odds ratio; TSDDs: Total standardized daily doses.

**Table 5:** Association between benzodiazepine use and AD/dementia risk.

Study Type	Population Sample	Effect on Dementia/AD Risk	Statistical Data	Ref.
Meta-Analysis	*n* = 45,391	Long-term BZD users are at a higher risk of developing dementia in comparison to those who have never used the medication	RR: 1.49,95% CI: 1.30–1.72) forever user, RR:1.55 (95% CI 1.31–1.83) for recent users, and RR:1.55 (95% CI 1.17–2.03) for past users.	[101]
Longitudinal Study	*n* = 668 (aged ≥ 75)	Significantly lower incidence of AD in the BDZ+ group		[110]
Population-based Study German population	*n* = 105,725 (aged ≥ 60)	Increased dementia risk	OR: 1.21 (95% CI: 1.13–1.29, *p* < 0.001)	[97]
Three-city French population-based study	aged ≥ 65 yrs	Linked to lower cognitive ability but not to faster cognitive deterioration as one ages	Chronic use significantly associated with a lower latent cognitive level (β = −1.79 SE = 0.25 *p* ≤ 0.001) no association was found between chronic use and an acceleration of cognitive decline, (β × time = 0.010 SE = 0.04 *p* = 0.81),	[102]
Retrospective Cohort Study—US	*n* = 528,006 (aged ≥ 65 yrs)	Slightly raised the risk of dementia compared to not using them, but the risk did not significantly increase with higher exposure levels.	aHR: 1.06 (95% CI: 1.02–1.10) for low BZD exposure, 1.05 (95% CI 1.01–1.09) for medium BZD exposure, and 1.05 (95% CI 1.02–1.09) for high BZD exposure.	[109]
Umbrella Review of Meta-Analyses (5) and systematic review (15)		Weak evidence of dementia link	aRR: 1.06 (low users), 1.05 (medium users), 1.05 (high users)	[108]
Case–control study Canadian population	*n* = 38,741 (age ≥ 65 yrs)	Increased risk of AD	aOR: 1.51, 95% CI: 1.36 to 1.69;	[94]
A Systematic Review and Meta-Analysis (Asian, North American, European population)		Increased risk of dementia	Higher association in Asian (OR 2.40; 95% CI 1.66–3.47); moderate association in North American (OR 1.49; 95% CI 1.34–1.65) and In European (OR 1.43; 95% CI 1.16–1.75)	[95]
Matched cohort study Finnish population	*n* = 70,718	Increased risk of death	aHR = 1.4, 95% CI: 1.2–1.6	[96]
A Systematic Review and Meta-Analysis	*n* = 981,133 (in the systematic review) and *n* = 980,860 (in the meta-analysis)	Can be a risk factor for developing dementia	OR: 1.38, 95% CI: l 1.07–1.77	[98]
A Systematic Review and Meta-Analysis	*n* = 159,090	Increase dementia risk	OR: 1.39, 95%, CI: 1.21–1.59	[99]
Case–Control Study Swiss population	*n* = 2876 (Mean age 80 ± 7.5 yrs)	No association with increased AD risk	Long-term benzodiazepine use (≥30 prescriptions) aOR: 0.78 (0.53–1.14).	[105]
UK-based case–control analysis	*n* = 26,459 (aged ≥ 65 yrs)	No association with increased AD or VD risk	AD: aOR: 0.69 (0.57–0.85) or VD: aOR: 1.11 (0.85–1.45).	[106]
Analytical prospective nested case–control study Spanish population	*n* = 77,609	Increased AD risk	OR = 1.05, 95% CI: 1.01–1.10	[107]

AD: Alzheimer’s disease; aHR: Adjusted hazard ratio; aOR: Adjusted odds ratio; aRR: Adjusted risk ratio; CI: confidence interval; HR: Hazard ratio; RR: Risk ratio; OR: Odds ratio; VD: Vascular dementia.

**Table 6:** Association between PPI use and AD/dementia risk.

Study Type	Population Sample	Effect on Dementia/AD Risk	Statistical Data	Ref.
Prospective cohort study—Germany	*n* = 73,679 (aged ≥ 75 yrs)	Increased dementia risk	HR: 1.44, 95% CI: 1.36–1.52; *p* < 0.001	[120]
Prospective cohort study—US	*n* = 3484 (aged ≥ 65 yrs)	Not associated with dementia risk, even for people with high cumulative exposure	Dementia (*p* = 0.66) AD (*p* = 0.77)	[134]
Retrospective study— Veteran population, US	*n* = 23,656	Significant association	OR: 1.55	[121]
Nationwide cohort study—Taiwan	*n* = 1,000,000 (aged ≥ 65 yrs)	Increased dementia risk	aHR: 1.42; 95% CI, 1.07–1.84	[122]
Meta-Analysis	*n* = 642,305	No significant AD or dementia risk	Dementia: HR: 1.04 (95% CI: 0.92–1.15) AD: HR: 0.96 (95% CI: 0.83–1.09 *p* < 0.001)	[10]
Longitudinal, multicenter cohort study— Germany	*n* = 3327 (aged ≥ 75 yrs)	Increased risk of dementia and AD	Dementia: HR: 1.38 (95% CI: 1.04–1.83) AD: HR: 1.44 (95% CI: 1.01–2.06)	[123]
Comprehensive Review (8 systematic reviews, 1 clinical trial, 15 observational studies, 3 case–control studies, and 1 cross-sectional observational study)		No link to cognitive decline or dementia		[136]
A Nationwide Cohort Study—Taiwan	*n* = 15,726 (aged ≥ 40 yrs)	Increased dementia risk	aHR: 1.22; 95% CI: 1.05–1.42	[125]
Finnish Nationwide nested case–control study	*n* = 282,858	No clinically meaningful association between PPI use and the risk of AD Higher dose was not associated with an increased risk	1–3 years of use: aOR: 1.01, 95% CI: 0.97–1.06; ≥3 years OR: 0.99, 95% CI: 0.94–1.04. ≥1.5 defined daily doses: aOR: 1.03, 95% CI: 0.92–1.14	[130]
Observational, longitudinal study	*n* = 12,416 (aged ≥ 50 yrs)	Not associated with a greater risk of dementia or AD	Decline in cognitive function HR = 0.78, 95% CI = 0.66–0.93, *p* = 0.005 Conversion to MCI or AD (HR = 0.82, 95% CI = 0.69–0.98, *p* = 0.03). Intermittent use was also associated with a lower risk of decline in cognitive function (HR = 0.84, 95% CI = 0.76–0.93, *p* = 0.001) and risk of conversion to MCI or AD (HR = 0.82, 95% CI = 0.74–0.91, *p* = 0.001)	[131]
Population-based study—UK	*n* = 3,765,744 (aged ≥ 55 yrs)	PPI use was associated with decreased dementia risk	HR: 0.67, 95% CI: 0.65–0.67, *p* < 0.01.	[132]
A systematic review, meta-analysis, and bias analysis from 9 observational cohorts	*n* = 3,302,778	No clear evidence	Dementia: RR: 1.15 (95% CI = 1.00–1.31); AD: RR: 1.13 (95% CI = 0.93–1.38)	[133]
Community-based retrospective cohorts	*n* = 135,722 (aged ≥ 45 yrs)	AD was not higher among PPI users, and a slight increase in the risk of non-AD dementia was observed	AD: OR: 1.47; 95% CI: 1.18–1.83 non-AD dementias OR: 1.38; 95% CI: 1.12–1.70	[135]
Meta-analysis location (European, North American, Asian)	*n* = 1,251,562 (mean age ≥ 70 yrs)	No association found	HR = 0.98, 95% CI: 0.85–1.13	[137]
Large placebo-controlled randomized trial	*n* = 17,598 (mean age ≥ 65 yrs)	No negative effects related to the use of pantoprazole over 3 years, except for a potentially higher chance of contracting enteric infections	OR: 1.15; 95% CI: 0.89–1.50; *p* = 0.28	[138]

AD: Alzheimer’s disease; aHR: Adjusted hazard ratio; CI: confidence interval; HR: Hazard ratio; RR: Risk ratio; SHR: Sub-distribution hazard ratio; MCI: Mild cognitive impairment; OR: Odds ratio.

**Table 7:** Association between statin use and AD/dementia risk.

Study Type	Population Sample	Effect on Dementia/AD Risk	Statistical Data	Ref.
Time-varying status of statin use along with the dose–response relationship Korean population	*n* = 119,013 (≥60 years old) Statin users duration < 540 days	Short-term use linked to increased AD risk; consistent use decreased AD risk	aHR = 1.04; 95%, CI = [0.99–1.10]. Having at least 540 days of statin prescription and a cumulative defined daily dose of at least 540 were linked to a reduced risk of AD [aHR (95% CI) = 0.87 (0.80–0.95) and 0.79 (0.68–0.92), respectively].	[150]
Meta-Analysis (30 studies)	*n* = 9,162,509	Statin use is associated with reduced risks of dementia, AD, and vascular dementia (VD)	Dementia risk: RR:0.83, 95% CI: 0.79–0.87 AD risk: RR: 0.69, 95% CI: 0.60–0.80, *p* < 0. 0001), VD risk: RR: 0.93, 95% CI: 0.74–1.16, *p* = 0.54).	[151]
Systematic review of randomized clinical trials (3) and observational studies (21)	*n* = 1,404,459 (aged ≥ 60 yrs)	No adverse cognitive effects	OR: 1.03 [0.82–1.30] and OR: 1.0 [0.61–1.65]	[152]
Nationwide Cohort Study Taiwan	*n* = 33,398 (aged ≥ 60 yrs)	Reduction in dementia risk in older adults	Dementia: ([HR], 0.78; 95% CI, 0.72–0.85, *p* < 0.001).	[155]
Population-based study—Rotterdam	*n* = 6992	Reduction in dementia risk for both lipophilic and hydrophilic statins	AD: HR: 0.57; 95% CI: 0.37–0.90), HRs were equal for lipophilic (HR: 0.54; 95% CI: 0.32- 0.89) and hydrophilic statins (HR: 0.54; 95% CI: 0.26–1.11).	[151]
A cohort of older patients receiving polypharmacy	*n* = 29,047 patients exposed to polypharmacy (mean age 76 yrs)	Stopping statins while continuing other medications led to a rise in the long-term chances of experiencing cardiovascular events, both fatal and nonfatal	In contrast to those who continued the medication, patients who stopped taking it had a higher risk of hospitalizations due to heart failure (HR: 1.24; 95% CI: 1.07–1.43) and any cardiovascular events (HR: 1.14; 95% CI: 1.03–1.26), as well as all-cause mortality (HR: 1.15; 95% CI: 1.02–1.30) and emergency admissions for any reason (HR: 1.12; 95% CI: 1.05–1.19).	[159]
Population-based retrospective cohort study—Hong Kong	*n* = 104,295 (mean age 74.2 ± 13.6 yrs)	Reduced AD, VD, and dementia risk	Dementia: (multivariable-adjusted SHR 0.80, 95% CI 0.76–0.84, AD: (SHR 0.72, 95% CI 0.63–0.82), VD: (SHR 0.82, 95% CI 0.70–0.95), unspecified dementia: (SHR 0.80, 95% CI 0.75–0.85).	[160]
Nationwide Prospective cohort study— Danish population	*n* = 674,900 (age > 40 yr)	Increased CVD risk	During follow-up, the Hazard ratios for individuals with vs. without early statin discontinuation were 1.26 (1.21–1.30) for myocardial infarction and 1.18 (1.14–1.23) for death from cardiovascular disease.	[142]
Systematic review (60 case reports)	Mean age 62 yrs	Conflicting results		[141]
Community-based prospective cohort study	*n* = 2356 (age > 65 yrs)	No significant association	ACD: HR:1.33, 95% CI: 0.95–1.85 AD: HR: 0.90, CI: 0.54–1.51 An analysis of a specific subset of participants who had at least one APOE-ε4 allele and joined the study before age 80 revealed aHR: of 0.33, CI: 0.10–1.04.	[143]

AD: Alzheimer’s disease; aHR: Adjusted hazard ratio; ACD: awareness of cognitive decline; CI: confidence interval; CVD: Cardiovascular disease; HR: Hazard ratio; RR: Risk ratio; SHR: Sub-distribution hazard ratio; OR: Odds ratio; VD: Vascular dementia.

## Data Availability

Data are contained within the article.
